# Real-Time Detection of Seven Phases of Gait in Children with Cerebral Palsy Using Two Gyroscopes

**DOI:** 10.3390/s19112517

**Published:** 2019-06-01

**Authors:** Ahad Behboodi, Nicole Zahradka, Henry Wright, James Alesi, Samuel. C. K. Lee

**Affiliations:** 1 Biomechanics and Movement Science Program, University of Delaware, Newark, DE 19713, USA; ahadbeh@udel.edu (A.B.); nzahrad1@jhu.edu (N.Z.); 2 Department of Physical Therapy, University of Delaware, Newark, DE 19713, USA; henryw@udel.edu (H.W.); jfalesi@udel.edu (J.A.); 3 Shriners Hospitals for Children, Philadelphia, PA 19140, USA

**Keywords:** cerebral palsy (CP), functional electrical stimulation (FES), gait analysis, gait event, gait phase detection (GPD), gait pathology, motion capture

## Abstract

A recently designed gait phase detection (GPD) system, with the ability to detect all seven phases of gait in healthy adults, was modified for GPD in children with cerebral palsy (CP). A shank-attached gyroscope sent angular velocity to a rule-based algorithm in LabVIEW to identify the distinct characteristics of the signal. Seven typically developing children (TD) and five children with CP were asked to walk on treadmill at their self-selected speed while using this system. Using only shank angular velocity, all seven phases of gait (Loading Response, Mid-Stance, Terminal Stance, Pre-Swing, Initial Swing, Mid-Swing and Terminal Swing) were reliably detected in real time. System performance was validated against two established GPD methods: (1) force-sensing resistors (GPD-FSR) (for typically developing children) and (2) motion capture (GPD-MoCap) (for both typically developing children and children with CP). The system detected over 99% of the phases identified by GPD-FSR and GPD-MoCap. Absolute values of average gait phase onset detection deviations relative to GPD-MoCap were less than 100 ms for both TD children and children with CP. The newly designed system, with minimized sensor setup and low processing burden, is cosmetic and economical, making it a viable solution for real-time stand-alone and portable applications such as triggering functional electrical stimulation (FES) in rehabilitation systems. This paper verifies the applicability of the GPD system to identify specific gait events for triggering FES to enhance gait in children with CP.

## 1. Introduction

Motion capture (MoCap) is useful to objectively quantify human movement [1] and is often used clinically to analyze walking gait in individuals with cerebral palsy (CP) [2,3,4,5]. Complete gait analysis systems typically combine optical MoCap with force-sensing platforms (aka force plates) to collect kinematic and kinetic data, respectively. Typical gait has been described as a series of seven contiguous phases: Loading Response (LR), Mid-Stance (MSt), Terminal Stance (TSt), Pre-Swing (PSw), Initial Swing (ISw), Mid-Swing (MSw), and Terminal Swing (TSw) [2,3,6]. Detecting these phases during walking, known as gait phase detection (GPD), is a critical component of gait analysis. GPD can be used in conjunction with inverse dynamics to derive the forces and moments generated on the limbs during walking. While MoCap-based GPD (GPD-MoCap) is considered the gold standard [1,7,8,9,10,11], most GPD-MoCap systems are expensive and require extensive laboratory space, limiting their clinical utility [12].

Recent technological advancements in micro-electro-mechanical systems (MEMS) have given rise to a number of wearable, non-laboratory-based locomotion monitoring systems. Some MEMS devices have been used to evaluate the efficacy of mobility interventions for activities of daily living in able-bodied adults [7,13]. Other applications include human–machine interfaces [14,15], closed-loop control of rehabilitation robotics [7,16,17,18], and triggering muscle stimulation in functional electrical stimulation (FES) walking systems [19,20,21,22,23]. Typical requirements for these applications include cosmesis, portability, robustness, low cost and low power consumption [24]. Inertial sensors (aka inertial measurement units (IMU)), including accelerometers (which measure linear acceleration) and gyroscopes (which measure angular velocity), have been used extensively in the last decade for gait event detection [20,25,26,27]. The compact size and low power consumption of IMUs make them ideal for wearable motion-sensing applications and they have even been used as implanted devices [21]. Because they directly measure rotational motion—and because rotation about a joint is the locus of human locomotion [26]—gyroscopes are ideal for movement monitoring. Additionally, unlike accelerometers, gyroscopes are not affected by gravity and thus are less sensitive to placement—they provide nearly identical signals when mounted anywhere along the plane of interest [23]. Due to these advantages, and despite their higher power requirements [23], gyroscopes are typically preferred over accelerometers for GPD algorithms [25]. Gyroscopes have been evaluated for use in ambulatory GPD [12,20], calculation of spatio-temporal parameters of gait [26], and FES system control [20,21,22].

Most control strategies utilizing either rehabilitation robotics [7,25] or FES walking systems [19,20,22] are gait phase dependent and thus require accurate real-time GPD [7]. GPD algorithms must execute quickly to minimize gait event detection latency and, ideally, operate on minimal sensor data. Few sensor-based GPD systems are able to detect all seven gait phases in real-time (full-resolution) [19,20,21,22,28] and some have other deficiencies [8,10]. For example, Senanayake and colleagues’ GPD system—although full-resolution—is comprised of a number of sensors (four force sensitive resistors (FSRs) and two inertial measurement units, each consisting of a three-axis accelerometer, magnetometer, and gyroscope) and the researchers did not report GPD time errors relative to GPD-MoCap [8]. The high number of sensors in their system complicates both control algorithms and system setup. Accelerometer-based control algorithms suffer from drift resulting from signal integration, limiting their utility in control systems [12,29] and FSRs have their own shortcomings: they are sensitive to placement [21], degrade in performance over time [23], may suffer mechanical failure [21,26,30], require specific instrumented footwear [21], and may be affected by irregular ground contact patterns in atypical gait [23,26]. These limitations with IMUs and FSRs prompted us to develop a simple two-gyroscope GPD system that detects all seven phases of gait [9].

Due to the high incidence of and cost associated with CP, improved rehabilitation strategies for this patient population are critical [10]. Although gait phases are considered an appropriate trigger to control stimulation delivery in FES walking systems [19,20,21], there is a paucity of GPD systems for this population [10,25]. In a novel offline approach, Lauer and colleagues [10] successfully detected all seven phases of gait in individuals with CP using previously collected electromyographic (EMG) signals from bilateral vastus lateralis. GPD time errors were as high as 113 ms relative to GPD-MoCap. The use of EMG signals for GPD is not without complications; data are sensitive to electrode placement and motion artifacts [7]. Additionally, if EMG-based GPD is used to control FES, complex signal processing is necessary to differentiate physiological EMG signals from muscle activation due to applied FES [31,32]. Finally, Lauer et al. did not evaluate the real-time performance of their GPD algorithm, limiting its potential for clinical use [10]. Although precise knowledge of detection time errors is crucial for reliable real-time GPD systems (especially in FES applications), few studies have accurately investigated GPD time errors relative to GPD-MoCap (which is considered the gold standard). Some studies used GPD algorithms based on force sensing resistors (FSR) to evaluate system performance [1,7,11,23,26,30]. FSR-based algorithms are referred to as GPD-FSR, and are generally considered to be inferior to GPD-MoCap [21].

**Figure 1:**
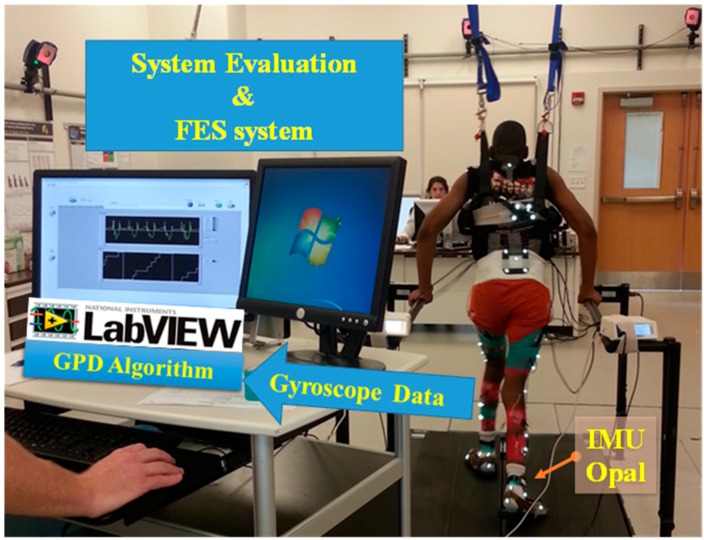
The gait phase detection (GPD) and functional electrical stimulation (FES) system. Shank attached gyroscopes sent data to a rule-based algorithm written in LabVIEW (version 2014, National Instruments, Austin, TX, USA) and all seven phases of gait were detected. A motion capture system was used to evaluate the system.

The aim of this paper is to adapt an existing real-time GPD system, designed to control multi-channel FES delivery for healthy adults during walking [9], for use with children with CP [33]. Because the GPD algorithm developed for use with healthy adults can also be used with typically developing (TD) children, this algorithm is referred to as GPD-TD. The GPD-TD system features: (1) a dual-gyroscope sensor setup, (2) the capability to detect all seven phases of gait, and (3) low detection time error. Gait phase onset detection times were previously evaluated on healthy adults [9]. This paper evaluates the performance of GPD-TD on typically developing children by comparing GPD data to GPD-FSR and GPD-MoCap. GPD-TD was modified to accurately detect gait phases for children with CP (GPD-CP) and re-evaluated relative to GPD-MoCap. The ultimate goal of this work is to use GPD-CP as a finite-state controller for a multi-channel FES delivery to promote more efficient gait patterns in children with CP (Figure 1). The use of GPD-CP in this manner is discussed in Behboodi et al. [33] and Zahradka et al. [34], a companion to the current paper.

## 2. Materials and Methods

### 2.1. Participants

**Figure 2:**
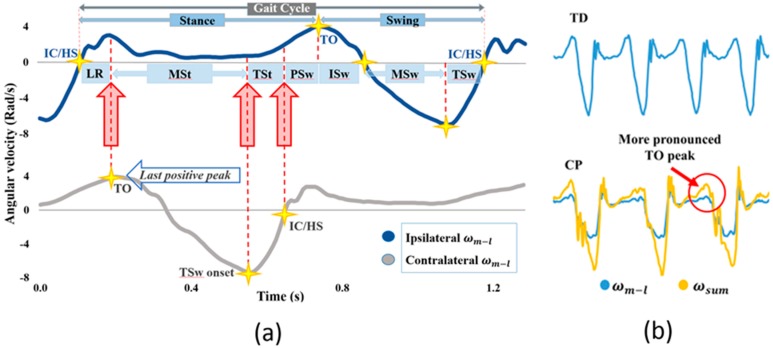
(**a**) Shank angular velocity about the medio-lateral axis ($\omega_{ml}$) for typically developing children and healthy adults during treadmill walking. Gait phase onset is based on the indicated peaks and zero-crossings. Four gait phase events are detected using ipsilateral shank angular velocity (loading response (LR), initial swing (ISw), mid-swing (MSw) and terminal swing (TSw). The three remaining gait phase events are detected using contralateral shank angular velocity (mid-stance (MSt), terminal stance (TSt) and pre-swing (PSw)). The onset of LR corresponds to initial contact (IC)/heel strike (HS) and the onset of ISw corresponds to toe-off/end-contact (TO/EC); (**b**) Representative shank angular velocity about the medio-lateral axis for a typically developing child (top) and a child with cerebral palsy (CP) (bottom). A distinct peak is visible at the onset of ISw (TO/EC) in TD while the peak is less distinct in CP. The sum of all three components of shank angular velocity ($\omega_{sum}$) shows a more distinct peak at ISw onset, and was initially used for ISw detection in children with CP (bottom).

Seven typically developing children (12 ± 1 years of age) and five children with spastic diplegic CP (14 ± 1 years of age, gross motor function control system (GMFCS) Level II and III) participated in this study (Table 1). All participants with CP exhibited crouch gait. Participants walked on an instrumented treadmill (Bertec, Columbus, OH, USA) at their self-selected walking speed and were instructed to use handrails that were attached to the treadmill. Handrail forces were measured, but technical difficulties rendered these data unusable. Participants with CP were part of a study investigating an FES treadmill intervention for GMFCS Level II and III children with CP [34,35]. Although abnormal gait complicates gait phase identification (for example, there is no identifiable heel strike with equinus gait), the use of shank angular velocity in the sagittal plane (i.e., the medio-lateral component of shank angular velocity ($\omega_{ml}$)) obviates this issue: shank angular velocity still shows characteristic peaks, valleys and zero-crossings despite abnormal gait patterns (Figure 2) Study procedures were approved by Shriners Hospitals for Children-Philadelphia (Western IRB #2059). Consent and assent were obtained from participants.

### 2.2. Gait Phase Detection for Healthy Subjects 

For healthy adults and TD children, medio-lateral shank angular velocity ($\omega_{ml}$) has a definitive pattern during the gait cycle [11]. The typical pattern is three positive peaks in the stance phase followed by a deep negative peak in the swing phase (Figure 2a). This pattern, detected by a shank-attached IMU (APDM Inc., Portland, OR, USA), is the input signal to GPD-TD. One IMU was worn on the lateral side of each shank. Each IMU contained three triple-axis sensors: an accelerometer, a gyroscope and a magnetometer. Only the gyroscope signals were used with GPD-TD. With the IMU’s *z*-axis aligned in the medio-lateral direction (i.e., axis of rotation of the knee in the sagittal plane), the *z*-component of the gyroscope data ($\omega_{ml}$) was used. The IMUs were aligned such that knee flexion and extension resulted in positive and negative values of $\omega_{ml}$, respectively. The IMUs wirelessly streamed data to an access point (APDM Inc., Portland, OR, USA), which was connected to a desktop computer (Dell, Round Rock, TX, USA) via USB 2.0. Data was sampled at 128 Hz. A rule-based algorithm (Table 2), written in LabVIEW (version 2014, National Instruments, Austin, TX, USA), used $\omega_{ml}$ to detect all seven phases of gait as described in Behboodi et al. (the rules used for TD children in this study are identical to the rules used for healthy adults in Behboodi et al.) [9].

### 2.3. Tunable Parameters

There are four tunable peak detection parameters in the GPD algorithm; window width and peak threshold for both ISw and TSw (for more information, refer to LabVIEW documentation on point-by-point peak finding). For GPD-TD, window widths were set to seven samples for both ISw and TSw and peak thresholds were set to 2.5 rad/s and −4 rad/s, respectively.

### 2.4. Gait Phase Detection in Children with CP

The GDP-TD algorithm was tested on children with CP. Although children with CP do not often exhibit typical gait events (e.g., those with equinus gait may lack heel strike), shank angular velocity shows similar features (Figure 2) and can still be used to determine gait phases. However, some modifications to the GPD algorithm were necessary for GPD-CP. In particular, while $\omega_{ml}$ typically has easily identifiable peaks and zero-crossings (Figure 2a), the lack of a distinct peak at toe-off/end-contact (TO/EC) (Figure 2b) confounded ISw detection for children with CP. This issue was mitigated by using the arithmetic sum of all three components of shank angular velocity ($\omega_{sum}$) instead of $\omega_{ml}$ to detect the TO/EC peak. The summed signal featured a more prominent peak at TO/EC, isolating it from spurious peaks present in the $\omega_{ml}$ signal. Because $\omega_{sum}$ slightly leads $\omega_{ml}$ in time and because the MSw zero-crossing closely follows TO/EC, $\omega_{sum}$ was also used to detect the MSw zero crossing. This reduced the chances of erroneously detecting MSw (in $\omega_{ml}$) before detecting ISw (in $\omega_{sum}$).

Even with the increased detection reliability of ISw with $\omega_{sum}$, extraneous peaks and zero-crossings due to spasticity resulted in false detections of ISw and MSw. To mitigate this, the following criteria were added: (1) ISw detection was blocked until at least 60% of the average gait cycle duration had elapsed since the last LR detection; (2) MSw detection was blocked until at least of 25% of the average of the last 10 gait cycle durations had elapsed since ISw; and (3) peak detection threshold values (paragraph 2.3) were set to 25% of the smallest ISw peak height and highest TSw valley depth observed over the first few gait cycles.

### 2.5. Auto-Thresholding

At first, the tunable parameters were computed manually for each participant by observing $\omega_{ml}$ and $\omega_{sum}$ over time for GPD-CP. It was later determined that the tunable parameters could be computed automatically. An adaptive algorithm was added, which computed both ISw and TSw thresholds based on average peak amplitudes over the past five gait cycles. As the subject walked, thresholds were automatically updated based on their gait profile. Automatic thresholding increased detection consistency and ISw onset detection for more severe atypical gait patterns to the point where it was no longer necessary to use $\omega_{sum}$ to detect ISw for children with CP. That is, adaptive thresholding allowed us to return to the use of $\omega_{ml}$ for detection of ISw. Automatic thresholding was not necessary for GPD-TD, as the thresholds indicated above were sufficient for all TD participants.

### 2.6. Real-Time GPD Simulator

The GPD LabVIEW program was adapted to operate on previously recorded gyroscope data loaded from a file. This allowed us to quickly test multiple variations of the GPD algorithm on a variety of walking gait profiles. Automatic thresholding for GPD-CP was developed and tested using this real-time GPD simulator. While our real-time GPD simulator did not operate on real-time gyroscope data, it maintained the real-time timing by reading in both gyroscope data and timing data and metering the gyroscope data based on recorded sample times. Thus, in contrast to the non-real-time GPD techniques used in other studies [10,23], in which algorithms operated on an entire time series all at once, our GPD simulator maintained the real-time nature of the original gyroscope data thus allowing us to design algorithms suitable for real-time operation.

### 2.7. System Evaluation

GPD-TD was evaluated relative to GPD-FSR while both GPD-TD and GPD-CP were evaluated relative to GPD-MoCap. Detection reliability was defined as the number of gait phases detected vs. the total number of gait phases detected by the reference system (GPD-FSR or GPD-MoCap) over all gait phases for all participants. Gait phase onset times were compared between the system under test and the reference system via both mean error and root mean square error (RMSE). Additionally, gait cycle duration and gait phase duration as a percentage of gait cycle duration were compared between the system under test and GPD-MoCap. Gait cycle duration was computed using two consecutive LR onsets and compared to gait cycle duration computed from GPD-MoCap. Gait phase duration percentage was computed by normalizing the gait phase duration to the gait cycle duration computed by the respective systems and averaged across participants for each group. For both GPD-FSR and GPD-MoCap, participants walked on an instrumented treadmill at their self-selected speed with motion sensors attached to each shank. Participants walked for about 30 s with both lower limbs instrumented. Because participants walked at a variety of speeds, instead of using data collected over a time duration, measures were computed using the last ten complete gait cycles. Participants were instructed to use handrails built into the treadmill. While GPD-MoCap can be challenging with participants with CP, the events indicated in Table 2 are universal enough to occur in many gait types. In particular, initial contact (IC) and end contact (EC) were used instead of toe-off and heel-strike, respectively.

#### 2.7.1. GPD-TD to GPD-FSR

For comparison with studies that used GPD-FSR as their reference system [5,11,21,30], the onset of LR (heel strike (HS) or IC), TSt (heel-off (HO)), and ISw (TO/EC) was compared between GPD-TD and GPD-FSR (Table 2) for seven TD participants (Table 1). FSRs (Interlink Electronics, Westlake Village, CA, USA; force range 0.18–20 N) were placed under the heel (FSR-Heel) and toe (FSR-Toe) of each foot and participants walked on the treadmill while both GPD-TD and GPD-FSR data were collected. Using a modified IMU (APDM Inc., Portland, OR, USA), FSR data were streamed to the GPD-TD computer. GPD-FSR data were processed in MATLAB (version 2015, The MathWorks Inc., Natick, MA, USA) as follows. The maximum FSR voltage observed during the swing phase was used as the baseline voltage; the FSR was considered to be off below baseline and on above baseline. The swing phase was roughly defined as the time between a sharp decrease in the FSR-Toe and a sharp increase in FSR-Heel. Reliability and mean onset error were computed.

#### 2.7.2. GPD-TD to GPD-MoCap

For evaluation versus the gold standard, GDP-TD was compared to GPD-MoCap. Seven TD participants (Table 1) walked on the treadmill with IMUs attached to each shank while MoCap (Motion Analysis, Rohnert Park, CA, USA) data were collected. Fifteen MoCap markers were placed on each lower extremity (medial and lateral femoral condyles (2), thigh cluster (4), medial and lateral malleoli (2), shank cluster (4), superior and inferior posterior calcaneus (2), medial first metatarsal head, lateral fifth metatarsal head) and seven on the pelvis (anterior superior iliac spines (2), midway between the posterior superior iliac spines, sacral cluster (4)). GPD-TD and GPD-MoCap were synchronized by triggering via COM port from the GPD-TD computer to the MoCap computer. The GPD-TD computer sent a trigger signal via the APDM access point to the COM port of the GPD-MoCap computer, remotely triggering data recording in the MoCap software (Cortex, Motion Analysis, Rohnert Park, CA, USA). Kinematic and kinetic data in the sagittal plane were analyzed in Visual 3D (C-Motion Inc., Germantown, MD, USA) (Figure 1). Reliability, onset, gait cycle and gait phase duration were computed and compared between the two systems.

#### 2.7.3. GPD-CP to MoCap

The above evaluation was repeated for five children with CP. Reliability, onset and gait phase duration were computed and compared between the two systems.

## 3. Results

### 3.1. GPD-TD vs. GPD-FSR

**Figure 3:**
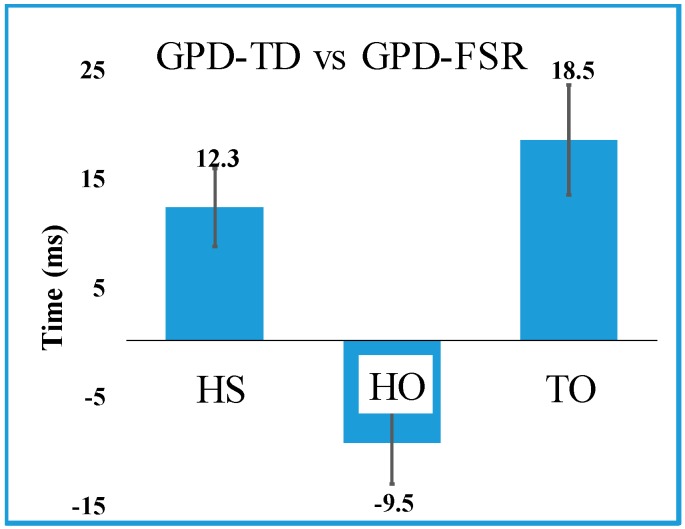
Mean error (± Std) of heel strike (HS), heel-off (HO) and toe-off (TO) for gait phase detection of typically developing children (GPD-TD) relative to gait phase detection via force sensing resistors (GPD-FSR). HS corresponds to loading response, HO corresponds to terminal stance and TO corresponds to initial swing.

GPD-TD was able to detect all events (LR onset (HS or IC), TSt (HO), and ISw onset (TO/EC)) detected by GPD-FSR for all participants over all gait cycles (100% reliability). Mean errors for all three events were less than 20 ms (Figure 3) LR onset (HS/IC) showed an error of 12.3 ms. TSt onset (HO) had the lowest error (−9.5 ms) while ISw onset (TO/EC) showed the highest mean error (18.5 ms). Note that negative deviations indicate delays with respect to GPD-FSR.

### 3.2. GPD-TD vs. GPD-MoCap

**Figure 4:**
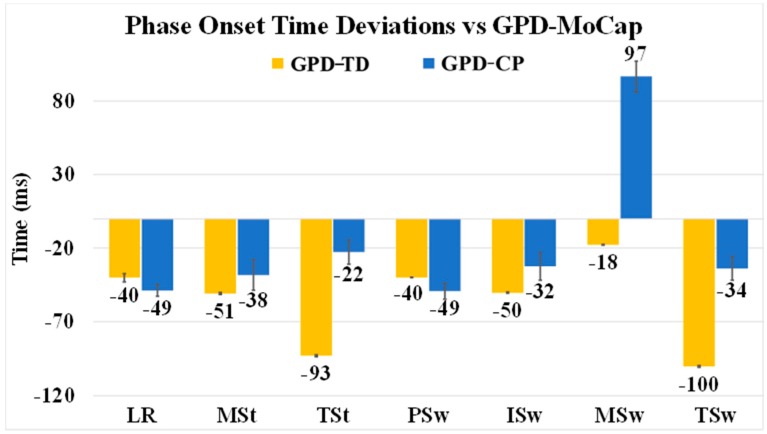
Gait phase detection (GPD) onset deviations (mean ± SE) relative to motion capture (GPD-MoCap). Deviations are shown for both the typically developing version of the GPD algorithm (GPD-TD, yellow) and for the version of the GPD algorithm used for participants with CP (GPD-CP, blue). Negative values indicate delays relative to GPD-MoCap.

**Figure 5:**
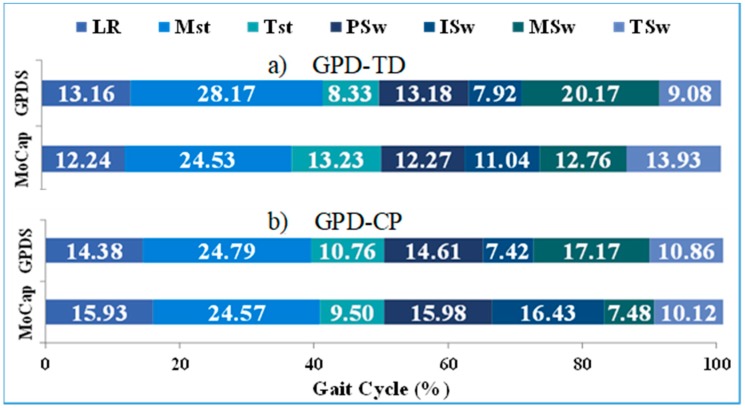
Gait phase duration relative to motion capture (MoCap) as a precentage of gait cycle for (**a**) CP gait phase detection (GPD) and (**b**) typically developing (TD) GPD. Each color indicates a gait phase, i.e., loading response (LR), mid-stance (MSt), terminal stance (TSt), pre-swing (PSw), initial swing (ISw), mid-swing (MSw) and terminal swing (TSw).

GPD-TD was able to detect 979 out of 980 events detected by GPD-MoCap for 99.9% detection reliability. Except for TSw and TSt (contralateral TSw), mean onset errors were below 51 ms. Minimum onset error was −18 ms (MSw) and maximum was −100 ms (TSw) (Figure 4). Onset RMSE ranged from 35 ms (MSw) to 105 ms (TSw) (Table 3). Note that negative deviations indicate delays in onset detection relative to GPD-MoCap. Average gait cycle duration RMSE was 22 ms (Table 4). MSw showed the highest difference between GPD-TD and GPD-MoCap (20.76% vs. 12.76%). Gait phase duration deviations relative to GPD-MoCap as a percentage of gait cycle can be seen in Figure 5. LR and PSw durations were close GPD-MoCap while TSw, MSw and TSw showed greater differences.

### 3.3. GPD-CP vs. GPD-MoCap

**Figure 6:**
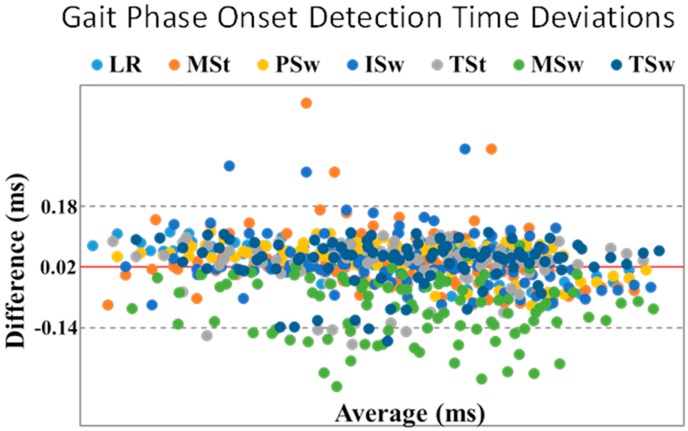
Bland–Altman plot for phase onset detection between CP gait phase detection (GPD) and motion capture (MoCap). Limits of agreement (gray dashed line) are the averaged difference (red line) ± 2 SD. Each color represents a gait phase, i.e., loading response (LR), mid-stance (MSt), terminal stance (TSt), pre-swing (PSw), initial swing (ISw), mid-swing (MSw) and terminal swing (TSw). A total of 700 data points were used from both CP GPD and MoCap.

For GPD-CP, detection reliability relative to GPD-MoCap was 99.6% (697/700). With the addition of automatic thresholding, detection reliability was increased to 100% (700/700). GPD-CP onset was delayed for all gait phases except MSw (97 ms early) (Figure 4). Mean error was highest for MSw (97 ms) and lowest for TSt (−22 ms) for GPD-CP. Onset RMSE ranged from 63 ms (LR) to 127 ms (MSw) (Figure 4). While the addition of automatic thresholding increased detection reliability, onset errors were slightly increased with automatic thresholding. A Bland–Altman plot graphically depicts onset errors for each gait phase for each subject (Figure 6). If the phase onset detection time errors were within the 95% confidence interval (±1.96 SD) about the mean (considered perfect agreement), the detection method was considered valid [37]. Each color represents one of the seven gait phases. Approximately 670 of 700 (96%) detected phases were within the 95% confidence interval. Most phases outside the interval were MSw (green), although three ISw (blue) and MSt (orange), two TSt (gray) and one TSw (navy blue) were outside of the confidence interval. Mean gait cycle duration RMSE was 22 ms (Table 4).

Gait phase duration deviations relative to GPD-MoCap as a percentage of gait cycle can be seen in Figure 5. With the exceptions of ISw (7.42% vs. 16.43%) and MSw (17.17% vs. 7.48%), most phases showed similar durations compared with GPD-MoCap. However, when ISw and MSw are taken together, the duration of the two phases matched quite well between GPD-CP (24.59% and GPD-MoCap (23.91%).

## 4. Discussion

The GPD algorithms in this paper successfully detected all seven gait phases in seven typically developing children and five children with CP. Detection reliability relative to GPD-MoCap was 100% for both GPD-TD and (with automatic thresholding) GPD-CP over 980 and 700 gait phases, respectively. Both algorithms use only two gyroscopes, making the GPD system compact and lightweight with low power consumption. These characteristics make our system appropriate for controlling a multiple channel FES system for use in gait training [33]. The use of raw gyroscope data eliminates the error propagation of typical IMU-based systems cause by double-integration. This simplified sensor system that can be used in sophisticated control algorithms used with rehabilitation robots [18] and neuroprosthesis [10,20,25,38]. Auto-thresholding eliminates the need to manually set threshold levels. Auto-thresholding rules were based on results from only five participants with GMFCS Levels II and III. Further testing on different populations with larger sample sizes is necessary to determine universally valid auto-thresholding rules.

Among major studies, only Senanayake et al. [8] and Lauer et al. [10] detected all seven phases of gait, as defined by Perry [6] (Senanayake et al. did not report onset errors and Lauer et al. did not evaluate their GPD system in real-time). While Smith et al. [38] and Pappas et al. [20] reported onset error relative to GPD-MoCap and evaluated their GPD systems in real-time, their systems were limited to five (Smith) and four (Pappas) phases (Table 5). Senanayake et al. required the use of 12 sensors and 22 signals to process, which is not ideal for a streamlined setup [8]. Similarly, Pappas et al. required three FSRs and one gyroscope for each limb [20] and Smith et al. required three FSRs for each limb [38]. Because both systems used FSRs, they are vulnerable to mechanical failure and may not be robust enough for daily living applications. Most importantly, these systems may not be suitable for individuals with atypical foot contact. The EMG-based system described by Lauer et al. [10] used a minimum sensor set up consisting of only one EMG sensor on each side. However, EMG is prone to motion artifacts using EMG in conjunction with FES can result in high-intensity electrical artifacts.

GPD-TD onset errors relative to GPD-FSR were favorable compared other studies [11,21,24,30]. Onset errors for HS and TO (12.3 (12) ms and 18.5 (17) ms (Figure 3), respectively, were among the lowest reported (Table 6). GPD-TD mean onset error for HO was 9.5 (13) ms (other references did not report HO, TSt onset error). Only Catalfamo et al. [24] reported standard deviations lower than our GPD system, indicating consistency in error (which makes it easier to compensate for timing errors in real-time applications). Gait cycle duration RMSE was low between the participants for both GPD-TD and GPD-CP (13 ms to 38 ms (Table 4)). Even though the onset detection delays in some phases were as high as −100 ms, (TSw for GPD-TD), offsets in other areas decreased the total gait cycle duration error to 22 ms.

TSt is an important gait phase for most FES walking systems as it not only provides the appropriate posture for PSw [39,40], but its detection can help to predict PSw in a more sophisticated system with a delay compensation algorithm [34]. During TSt, ankle dorsiflexion reaches peak torque, and ankle plantar-flexors are at their highest level of activity as they contract eccentrically for push-off and limb advancement during swing [6]. This could enhance the performance of an assistive device by increasing propulsion needed in PSw to advance the center of mass forward.

Although phase onset detection error is important for evaluation of any real-time GPD system, only Hanlon et al. [41], Pappas et al. [20], Smith et al. [38] and Skelly et al. [19] evaluated their systems using GPD-MoCap. Note that our GPD-FSR comparison showed lower onset error for LR onset (12.3) than our GPD-MoCap comparison (−40 ms). The reported error depends on the system used as the standard, against which the system-under-test is compared. Since GPD-MoCap is more accurate than GPD-FSR, it is more appropriate to validate one’s system relative to GPD-MoCap [8,10,11]. Our results show that one can obtain seemingly better results with a worse evaluation method (GPD-FSR, in this case).

Lower extremity finite state control orthoses [18], prostheses [42], and sophisticated FES walking systems [10,20,25,33,38] can benefit from full-resolution GPD. However, most GPD systems (such as those that are FSR-based) cannot detect swing phases. Many GPD systems can only detect LR and PSw [7,22,26,30,43,44], making them inappropriate for applications requiring higher-resolution GPD.

Some studies evaluate detection of foot-flat (FF) [1,19,20,21,28,29]. FF, depending on the detection algorithm, is reasonably close to MSt onset, which can be detected using contralateral TO/EC [6]. Consequently, unless there is a need for unilateral detection, FF detection is unnecessary. In contrast, TSt onset (HO) detection may be relatively more important because, with the combination of this event with both LR onset (HS) and ISw onset (TO/EC) and use of a contralateral algorithm, one can detect all of the stance phases. It should be noted that comparison with GPD-FSR revealed that all the stance phases detected by the GPD system have relatively low onset detection time errors relative to other studies that evaluated their systems vs. GPD-FSR; absolute values of LR, TSt and ISw (HS, HO and TO/EC respectively) onset detection time errors were all below 20 ms.

After PSw, the rest of limb advancement and forward progression occurs during the swing phases [6] and three major muscle groups contribute to this end: Gluteus maximus (Glut), hamstrings (Ham), and quadriceps (Quad) activate during either MSw or TSw [45]. To slow down the rate of knee extension, Ham starts firing at MSw onset while Glut and Quad activate at the end of TSw onset to ensure full knee extension and to prepare for shock absorption at LR onset (HS) [6]. Consequently, to control the activation of these three muscles, in applications such as FES, detection of all swing phases is critical. As mentioned above, however, there are limited GPD systems capable of detecting these individual swing phases. Besides Lauer [10] and Senanayake [8], to the best of our knowledge, there are only two other well-cited detection systems capable of detecting any swing phases, and ISw was the only swing phase detected in both. Smith et al. [38] used an FSR insole in combination with a fuzzy classifier to detect ISw. Skelly [19], in addition to TO/EC (i.e., ISw onset), detected maximum knee flexion, which is close to the end of ISw based on Perry’s definitions. Our GPD system detected all the swing phases in real time using only one gyroscope sensor on each shank.

There were several limitations in the present study. Gait phase onset detection delay—for ISw, in particular—is sensitive to each participant’s ability to walk and advance their lower extremities. Participants who are less skilled at ambulation (i.e., higher level on the Gross Motor Function Classification System) showed higher onset detection root-mean-square errors (RMSE) in ISw, MSt (contralateral ISw (Table 2)) and MSw. The RMSE of phase detection onset for each participant with CP and their GMFCS level can be found in Appendix A. Although modeling ISw onset from the medio-lateral shank angular velocity (i.e., last positive peak of $\omega_{m-l}$ (Figure 2a)) is a well-established kinematic rule based on gait analysis studies such as Aminian et al. [26], Jasiewcz et al. [30], Monaghan et al. [22], Catalfamo et al. [24] and Lee et al. [11], its reliability may be suspect when used in populations with higher GMFCS levels. Thus, finding a robust ISw detection rule using $\omega_{ml}$ for children with higher GMFCS levels may require further investigation.

TSw and MSw detection need additional investigation because they showed inconsistency between groups. TSw had the highest onset detection delay in the TD group (−100 ms), whereas it was among the lowest (−34 ms) in the CP group. In contrast, MSw had the lowest onset detection error (−18 ms) in the TD group, but its error was the highest in CP group (97 ms) (Figure 4). Like most GPD studies, IC was not included as a gait phase. In Perry’s definition of gait, LR and IC are two separate phases [6]. Because IC is less than 10% of the gait cycle and its muscle activation pattern is the same as in LR, Perry also merged these two phases when demonstrating muscle activation during gait [6]. This merged approach is pervasive in GPD for FES applications [10,23,38]. As can be seen in the $\omega_{ml}$ pattern (Figure 2a), there are two peaks after negative to positive zero crossing (i.e., LR onset in the GPD system) that are potentially associated with the end of IC that might be used to isolate IC from LR. Further processing is needed to verify this assumption.

Determining sources of GPD onset errors can prove challenging. Once identified, compensating for them can be just as difficult. One major source of delay was the Windows Task Scheduler, which sometimes delayed the communication between the GPD computer and the MoCap computer. The GPD system’s raw signal was slightly delayed in all of the data sets (66 ± 1.6 ms) relative to GPD-MoCap. This was considered a major contributing factor in onset detection delay of the GPD system and should be addressed in future development.

There are some small but impactful improvements that can increase determinism and decrease onset detection time errors of the system. First and foremost, the system should be implemented in a real-time stand-alone platform, such as the National Instruments Compact-Rio, which is compatible with our LabVIEW based GPD algorithm. Substituting commercial IMUs with a customized gyroscope sensor may decrease data transmission latency associated with additional unused sensors onboard the IMU. Wireless signal transmission protocols used by the Opal IMUs produced additional signal delays and are prone to interference—use of a wired gyroscope may reduce latency and interference.

Exhaustive timing data was not collected, but average control loop iteration time—including gyroscope data acquisition, GPD computation, auto-thresholding and commanding the stimulators—was in the millisecond range. This is low enough to contribute virtually no timing error, given that Wi-Fi latency and latency due process pre-emption by the Windows Task Scheduler are typically much higher (later testing revealed intermittent spikes as high as 200 ms in iteration time) and that observed gait phases were no shorter than 50 ms. In addition, our results indicate that our system is reliable relative to MoCap. Nevertheless, further testing is necessary to determine if the system can be reliably used long-term with wireless sensors in a non-real-time operating system. If not, wired gyroscopes can be used to eliminate Wi-Fi latency and the software can be re-deployed onto a real-time operating system such as Real-Time Linux or into an embedded system such as the NI CompactRIO platform from National Instruments.

Other limitations of this study were the low number of participants (seven TD and five with CP), and the use of handrails during treadmill walking. Push-off force at EC was not computed due to technical difficulties with handrail force data; future studies will aim to correlate push-off force with therapeutic outcomes. Overhead harnesses were used for safety but were not observed to have significantly affected gait. While sufficient for our study, auto-thresholding may be improved by using an adaptive method similar to the Self-Tuning Threshold Method used in Tang [46], which would increase the sensitivity of the algorithm to rapidly changing gait. A larger sample of children with CP may have resulted in more robust automatic detection parameters that would apply to a greater population of children. A larger sample of TD participants may have revealed variability that would have necessitated the use of auto-thresholding for this population as well. Further research is necessary to develop automatic thresholding algorithms for general use in all populations.

## 5. Conclusions

The GPD system detected all seven gait phases in children with CP in real time, making it a viable option for controlling stimulation delivery in walking FES systems. A thorough understanding of detection errors relative to MoCap may result in development of a compensatory mechanism and increase the system’s potential for further development. Furthermore, its minimal sensor setup, using only one gyroscope on each side, makes it a good choice for portable real-time systems.

## Figures and Tables

**Table 1:** Participant age, gender, Self-selected walking speed (SSWS), Gross Motor Function Classification System (GMFCS) level, height and weight.

	Age (yrs)	Gender	SSWS (m/s)	GMFCS	Height (m)	Weight (kg)
TD01	16	M	0.8	N/A	1.78	71.92
TD02	10	M	0.8	N/A	1.46	32.55
TD03	10	F	1.2	N/A	1.46	31.95
TD04	12	F	1.25	N/A	1.59	43.25
TD05	12	F	1	N/A	1.47	36.42
TD06	14	F	1.1	N/A	1.55	52.61
TD07	13	F	1.1	N/A	1.73	56.29
CP01	15	M	0.6	III	1.67	32.13
CP02	16	M	0.8	III	1.70	60.06
CP03	18	M	0.9	II	1.70	61.97
CP04	12	M	0.75	II	1.52	41.50
CP05	13	F	0.8	II	1.45	81.49
**Mean**	**13.42**		**0.93**		**1.59**	**50.18**
**STD**	**2.36**		**0.19**		**0.12**	**15.85**

**Table 2:** Gait phase detection (GPD) events for the algorithm used in this paper (GPD-TD), motion-capture-based GPD (GPD-MoCap) and force-sensitive-resistor based GPD (GPD-FSR).

Gait Phase	GPD-TD Event (*ω_ml_*)	GPD-MoCap Event	GPD-FSR Event
LR Onset/HS/IC	Zero-crossing (negative to positive) [22]	IC on force plate [6]	Heel FSR on
MSt onset/FF	Contralateral TO [6]	Contralateral TO [6]	
TSt onset/HO	Contralateral TSw [6]	Contralateral TSw [6]	Heel FSR off
PSw onset	Contralateral IC/HS [6]	Contralateral IC [6]	
ISw onset/TO/EC	Last positive peak [30]	EC on force plate [6]	Toe FSR off
MSw onset	Zero-crossing (positive to negative)	Max knee angle [6]	
TSw onset	Valley [36]	Max shank angular velocity [36]	

Gait phases (loading response (LR), mid-stance (MSw), terminal stance (TSt), pre-swing (PSw), initial swing (ISw) and terminal swing (TSw) are based on kinematic or kinetic data. LR onset corresponds to heel strike (HS), also called initial contact (IC). MSw onset corresponds to foot-flat (FF). TSt onset corresponds to heel-off (HO). ISw onset corresponds to toe-off (TO), also called end-contact (EC). Events for GPD-TD are based on medio-lateral shank angular velocity ($\omega_{ml}$).

**Table 3:** Root mean square error (RMSE) of Gait phase detection (GPD) onset relative to motion capture (GPD-MoCap) for the typically developing version of the GPD algorithm (GPD-TD), and for the version of the GPD algorithm used for participants with CP (GPD-CP) without auto-thresholding (AT) and GPD-CP with AT. Times are in ms. Gait phases are loading response (LR), mid-stance (MSt), terminal stance (TSt), pre-swing (PSw), initial swing (ISw), mid-swing (MSw) and terminal swing (TSw). Times are in ms.

	LR	MSt	TSt	PSw	ISw	MSw	TSw
**GPD-TD**	52	70	98	52	70	35	105
**GPD-CP without AT**	63	96	69	63	81	127	70
**GPD-CP with AT**	63	88	84	55	88	141	89

**Table 4:** Gait cycle duration RMSE relative to motion capture for both the typically developing version of the GPD algorithm (GPD-TD) and for the version of the GPD algorithm used for participants with CP (GPD-CP). Times are in ms.

	Subject Number							Mean ± SE
	01	02	03	04	05	06	07	
**GPD-TD**	23	23	27	17	28	16	21	22 ± 1.7
**GPD-CP**	21	13	38	24	16	N/A	N/A	22 ± 4.3

**Table 5:** Comparison of our GPD system to similar GPD systems from well-cited studies. Gray cells indicate relative shortcomings of the corresponding system. The last row is our GPD system.

Study	No. of Detected Phases	Real Time	Sensor Setup on Each Side	Onset Detection Time Error Reported
Lauer et al. [10]	7	No	1 EMG	Yes
Senanayake et al. [8]	7	Yes	4 FSR + 6 Inertial sensors (2 IMU)	No
Pappas et al. [20]	4	Yes	3 FSR + 1 Gyro	Yes
Smith et al. [38]	5	Yes	3 FSR	Yes
Our GPD system	7	Yes	1 Gyro	Yes

**Table 6:** Mean and one standard deviation (SD) of calculated delays in studies that evaluated their gait phase detection (GPD) algorithm vs. force-sensing resistor GPD (GPD-FSR). Values are in milliseconds and negative values correspond to delays relative to GPD-FSR. Delays are reported for heel strike (HS) and toe-off (TO).

Study		Gait Events	
		HS Mean (SD)	TO Mean (SD)
Lee et al. [11]		19	−3
Kotiadis et al. [21]	System 1	~−40 (20)	~100 (35)
	System 2	~−60 (20)	~10 (25)
Catalfamo et al. [24]		−8 (9)	50 (14)
Jasiewiz et al. [30]	System 1	−11 (23)	19 (34)
	System 2	−12 (22)	15 (26)
	System 3	−14 (23)	23 (28)
Our GPD system		−12.5 (12)	−18.5 (17)

## Appendix A

**Table A1:** Root mean square error (RMSE) of gait phase detection onset relative to motion capture for five children with cerebral palsy (CP). Gross motor function control system (GMFCS) is indicated. Gait phases are loading response (LR), mid-stance (MSt), terminal stance (TSt), pre-swing (PSw), initial swing (ISw), mid-swing (MSw) and terminal swing (TSw).

ParticipAnt Number	GMFCS Level	Gait Phase						
		LR	MSt	TSt	PSw	ISw	MSw	TSw
1	3	81.6	162	52	82	127	196	58
2	3	68.9	124	84	70.0	133	155	77
3	2	38	48	50	38.7	49	95	56
4	2	58	20	80	60	18	71	73
5	2	59	39	71	59	39	60	80
